# Supplementary figures and images for: Classical mathematical models for prediction of response to chemotherapy and immunotherapy
Source: PLoS Comput Biol. 2022 Feb 4;18(2):e1009822. doi: 10.1371/journal.pcbi.1009822 (PMC8903251; doi:10.1371/journal.pcbi.1009822)

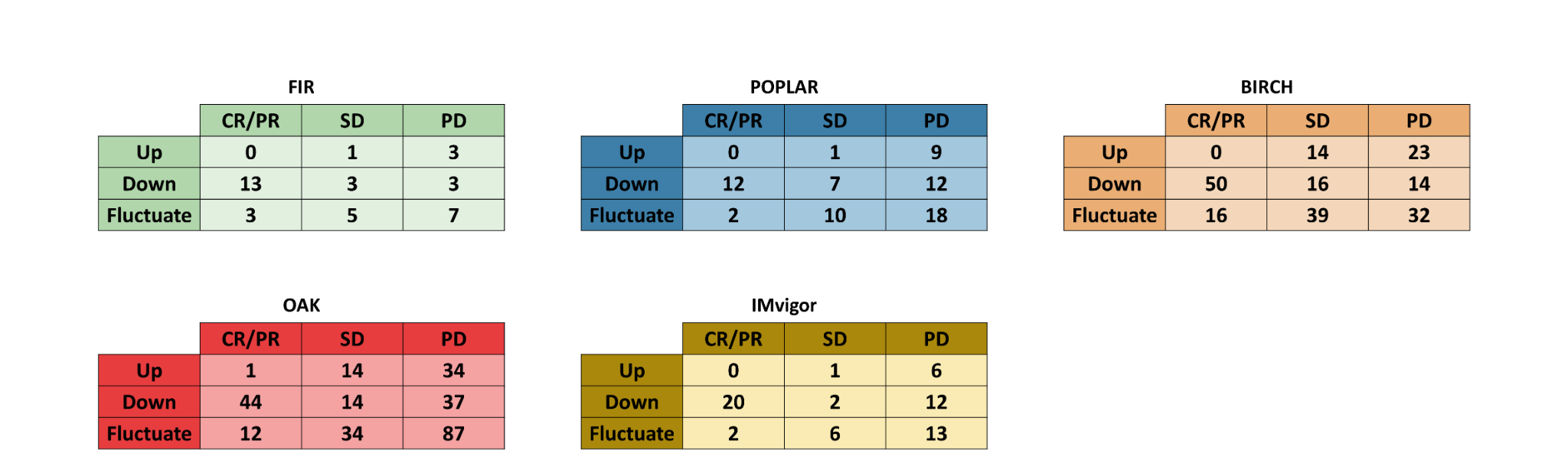

Supplement: S1 Fig — (TIF) [file pcbi.1009822.s001.tif]

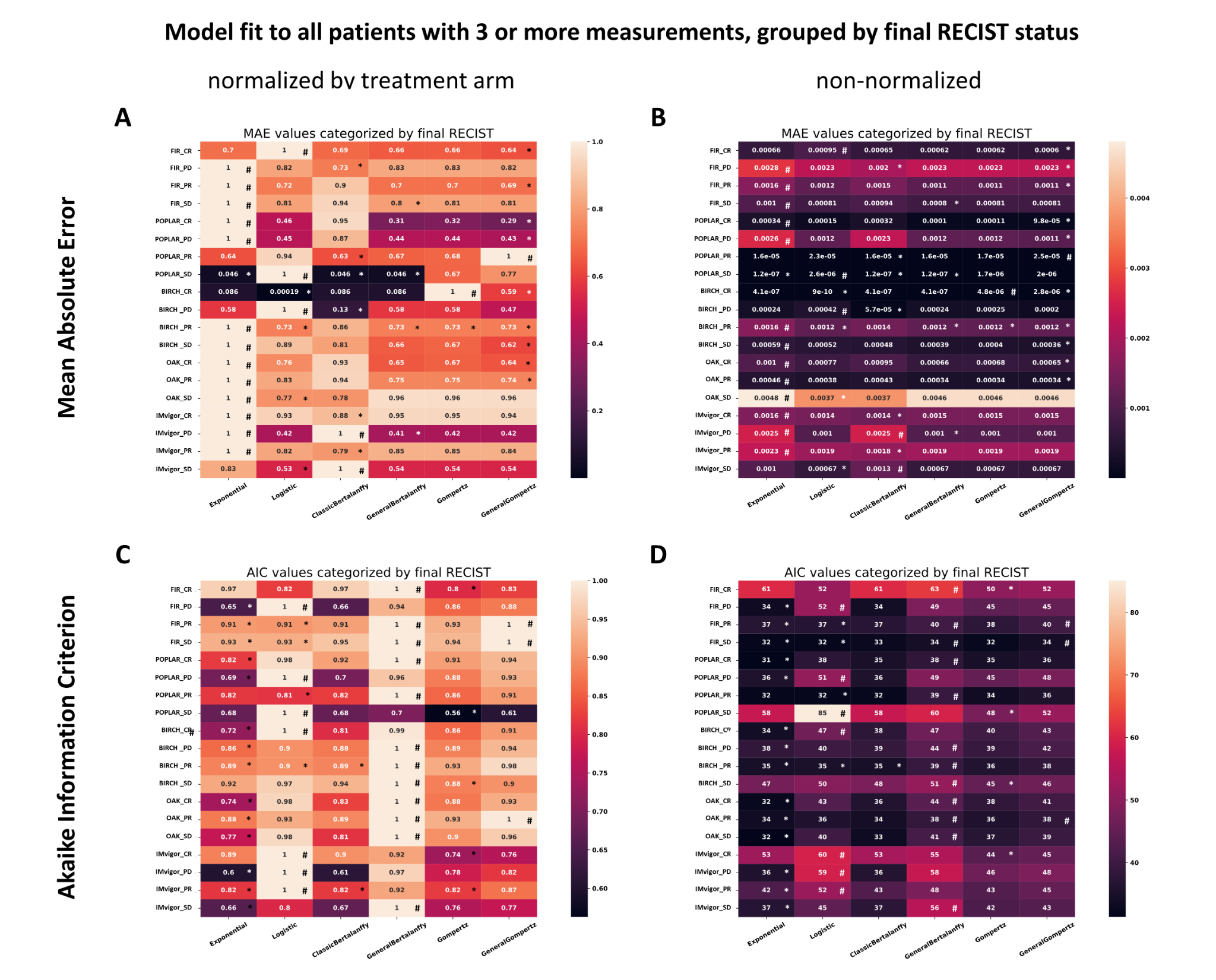

Supplement: S2 Fig — (A) Model fit for all treatment arms in all trials, stratified by final RECIST, for all models. The loss function is the Mean Absolute Error (MAE, L1-Loss), after row-wise normalization. (B) Corresponding plot without row-wise normalization, showing the raw MAE. The worst MAE in each figure is indicated with “#” and best one is indicated with “*”. (C) Corresponding plot showing the Akaike Information Criterion (AIC) which penalizes models with a large number of free parameters, row-wise normalized. (D) Corresponding plot without row-wise normalization. (TIF) [file pcbi.1009822.s002.tif]

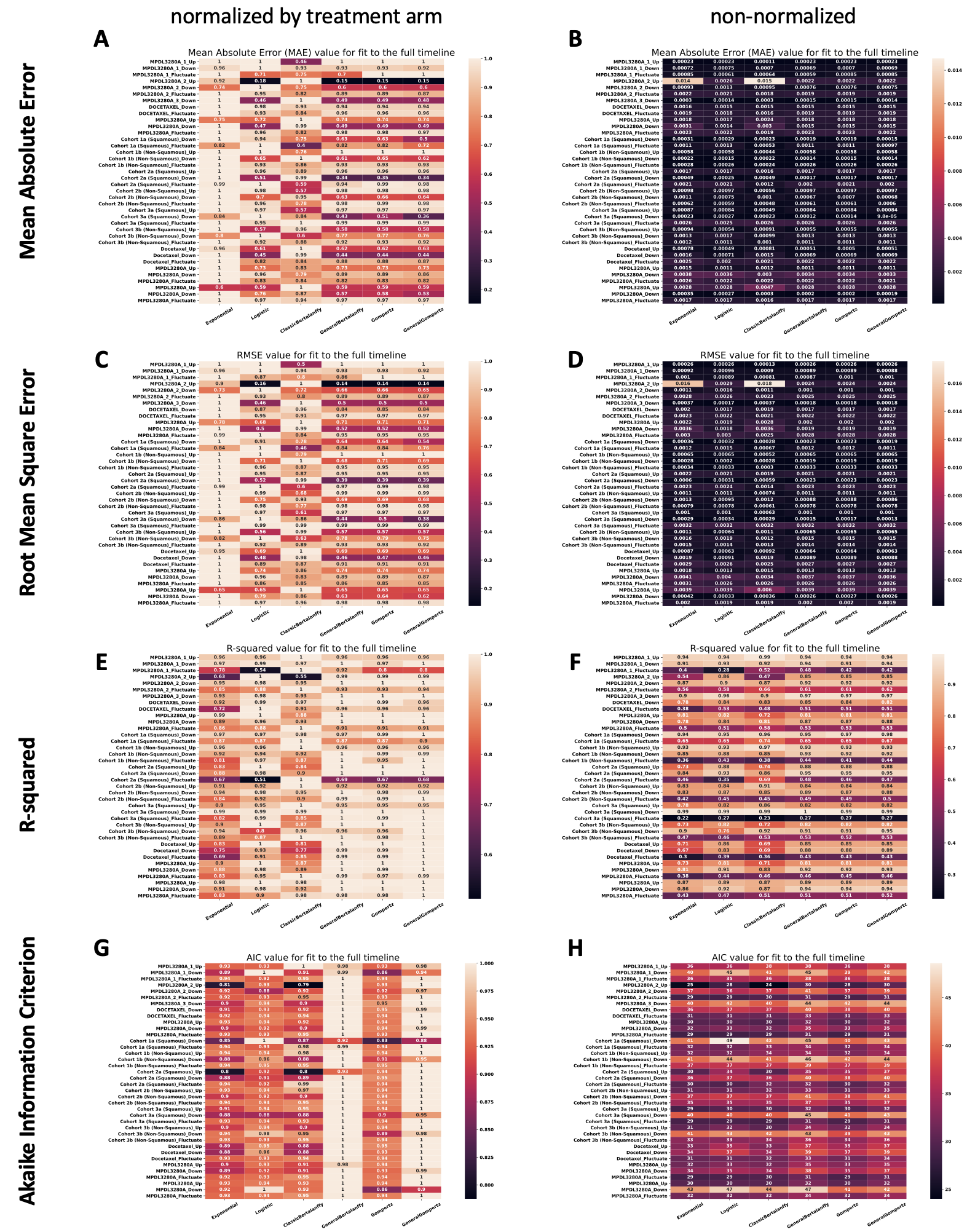

Supplement: S3 Fig — (TIF) [file pcbi.1009822.s003.tif]

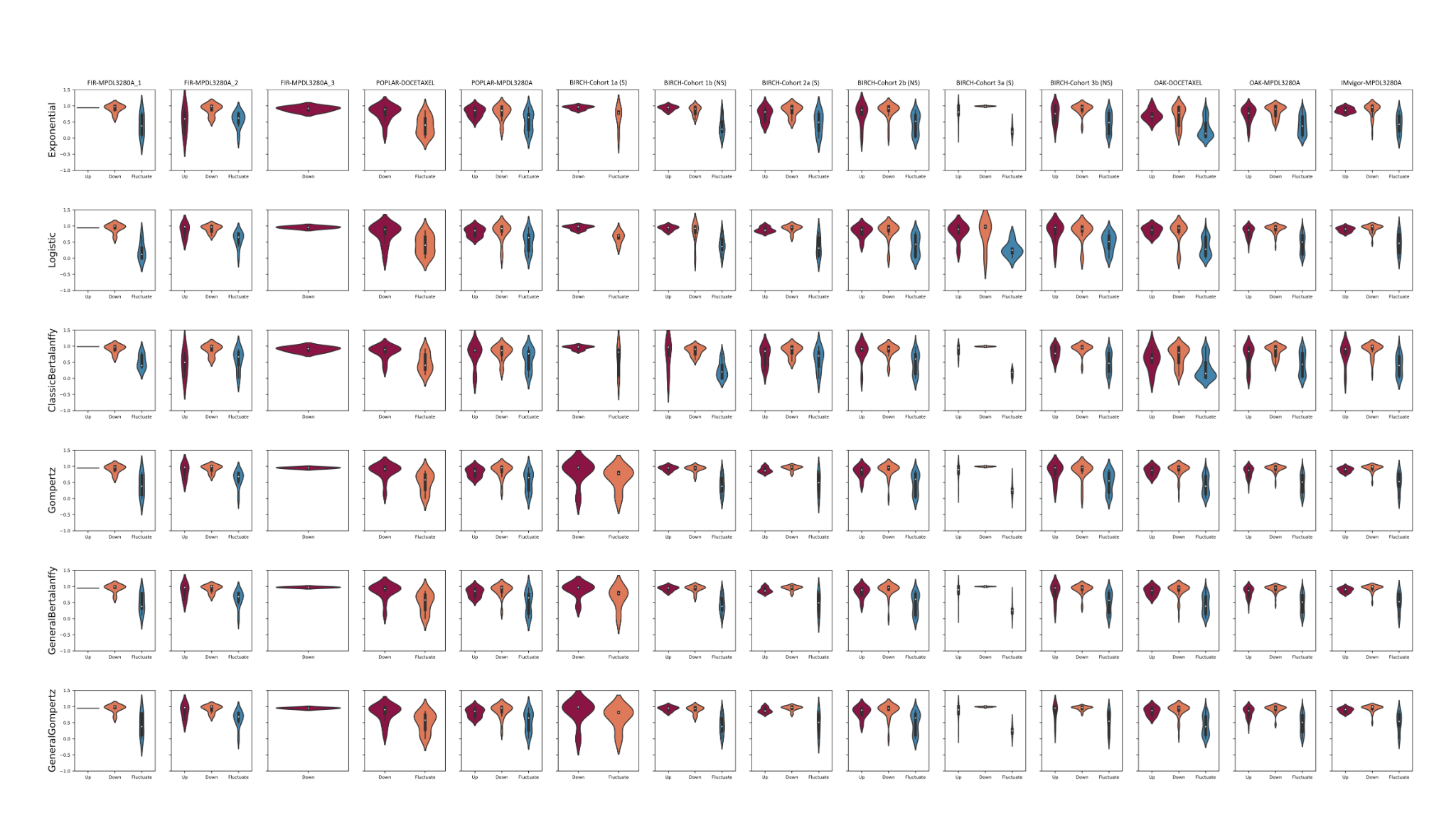

Supplement: S4 Fig — (TIF) [file pcbi.1009822.s004.tif]

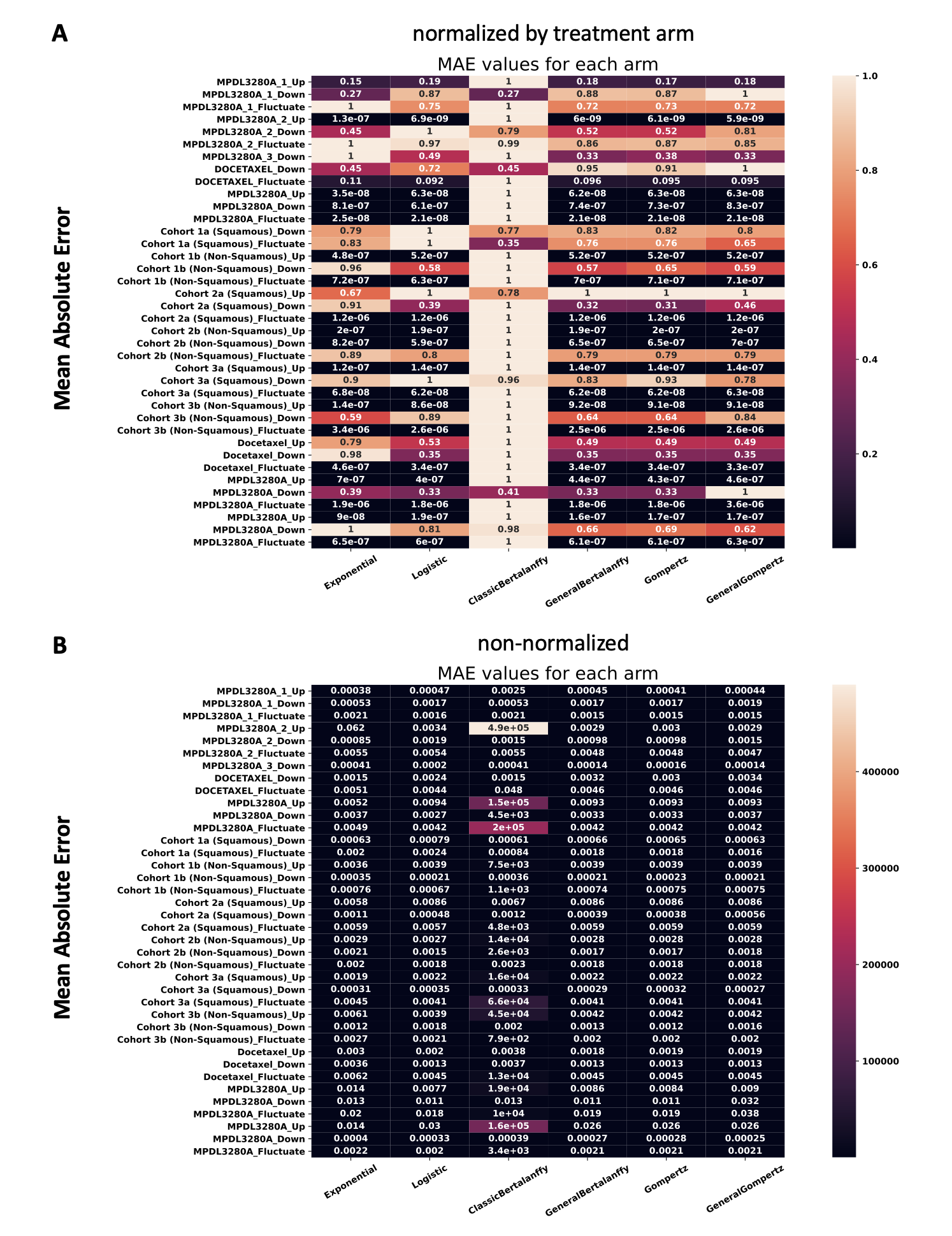

Supplement: S5 Fig — Results of experiment #2. (TIF) [file pcbi.1009822.s005.tif]
