# Supplementary material for: Classical mathematical models for prediction of response to chemotherapy and immunotherapy
Source: PLoS Comput Biol. 2022 Feb 4;18(2):e1009822. doi: 10.1371/journal.pcbi.1009822 (PMC8903251; doi:10.1371/journal.pcbi.1009822)
Supplement: S1 Text — This project proposal was submitted originally to the data sharing platform. (DOCX) [file pcbi.1009822.s008.docx]

S1 Text: Original data sharing request

## Proposal

Anti-PD1/PDL1 treatment has been approved in a number of tumor types and can induce durable responses in a subset of patients. It is a common clinical problem to identify these responders early during treatment. The temporal dynamics of response to anti-PD1/PDL1 treatment are different than for classical cytotoxic or targeted agents: Some patients respond immediately, some patients show a pseudoprogression followed by a response and some patients stay stable for a prolonged time and respond after months of treatment.

In our interdisciplinary group, we have previously developed computational models that can predict immunotherapy response in individual patients. These models can be calibrated to individual patients and can predict optimal treatment schedules and combinations (Kather et al., Cancer Research 2017 and Kather et al., Cancer Research 2018; PMID 29967263 and 28923860). These previously published models relied on spatial data of cells in the tumor microenvironment (TME) obtained via image analysis of histological slides.

We have now developed and validated non-spatial models with the aim of predicting response and scheduling optimal treatment plans based on non-spatial data, as explained below. In our preliminary experiments we have used these computational models in small cohorts of patients treated with immunotherapy in different tumor types. Our preliminary data show that these models can

1) identify non-response and response early during treatment

2) identify non-responders to anti-PD1/PDL1 treatment who could be rescued by adding anti-CTLA4 antibodies

3) predict the lymphocyte number in the tumor microenvironment non-invasively

This non-spatial model requires de-identified patient level data (tumor volume measurements over time, clinical outcome and, optionally, lymphocyte density in tumor tissue) as explained in section 3.1.

We are planning to validate our model retrospectively in patient cohorts who were treated with anti-PD1/PDL1 treatment and we kindly request access to this anonymized, readily available data from the selected previous clinical trials.

## Lay person summary

Immune checkpoint inhibitors can control, shrink or even cure immunogenic tumors such as melanoma, lung cancer or microsatellite instable gastrointestinal cancer. However, in practice, most patients with these tumors do not respond to immunotherapy alone. It is very difficult to predict who will respond and who will need an additional or alternative “rescue” treatment strategy. This is mainly because immunotherapy responses look very different than classical treatment responses in oncology: some patients immediately respond, some show a delayed response after weeks of treatment and some patients progress initially and respond afterwards. In short, linear thinking fails in these highly non-linear response patterns and clinical intuition can be misleading in predicting immunotherapy treatment outcome.

## Statistical analysis

We have developed and published computational models which can predict treatment response based on readily available clinical data for individual patients (PMID 29967263 and 28923860). These models can be categorized in two groups: Spatial models (agent-based model) which take into account the spatial layout of cells in the tumor and non-spatial models which neglect spatial arrangement of cells and assume a homogenous mixture of cells in the tumor. Both types of models are established in our group and both have advantages and limitations. Spatial models are more realistic and can yield 3D simulations of tumors which can be compared to actual imaging data. An example of a 3D simulation generated with our platform can be viewed here: https://www.youtube.com/watch?v=D1WoELrzIwc. In contrast, non-spatial models ignore the spatial layout of cells in the tumor. Instead, they keep track on the total number of cells for each cell type. Assuming that the tumor consists of billions of cells, these models assume that spatial effect are averaged out. While this is a strong simplification of reality, these models can be very useful. These non-spatial models can be expressed in terms of ordinary differential equations (ODEs), enabling a clear understanding of the system's dynamics.

In summary, we have established spatial and non-spatial computational models of tumor growth with or without immunotherapy. Previously, we have validated these models with our in-house data. Now, we would like to fit the non-spatial model to external data. Our main strategy is to fit the model to data up to a certain time point and then use the model to predict tumor response after that time point (see below). To do this, we request access to the following patient-level data:

1) multiple (at least four) measurements of tumor size at different time points during treatment

2) Optional: the number of tumor-infiltrating lymphocytes as measured histologically in biopsies or surgical tissue specimen.

3) For response prediction, information about the clinical outcome of the patients as assessed by RECIST

In the future, we will use this approach in adaptive clinical trials in which treatment strategies are optimized according to early treatment response dynamics. Thus, this model could be a useful tool for mathematically informed decision making in cancer immunotherapy.

## Hypotheses

We will to use the above-mentioned data to investigate two main hypotheses:

Hypothesis 1: A non-spatial computational model can predict response to Anti-PD1/PDL1 treatment based on tumor volume measurements over time.

Hypothesis 2: This model can predict intratumoral lymphocyte density from clinical response data.

We have previously shown this in a small patient cohort and would like to validate this in larger cohorts from multiple tumor types. This is a retrospective and exploratory analysis that will yield prediction that can be validated in prospective clinical trials. Also, we are planning to perform adaptive clinical trials to answer the following hypothesis:

Hypothesis 3: For non-responders, the model can predict who will benefit from additional treatments in addition to Anti-PD1/PDL1.

Although Hypothesis 3 cannot be answered with retrospective data from the above-mentioned trials, the present study will lay the groundwork to design clinical trials to test this hypothesis.

## Conclusion

Our model requires tumor volume measurements over time for patients treated with anti-PD1/PDL1. We will explore the robustness of the model in a wide range of diseases and will use this to generate a robust methodological framework for our planned prospective clinical trials. We request access to all six selected trials that include patients who have been treated with the anti-PDL1 antibody Atezolizumab.

In order to predict outcome from early treatment response dynamics, our non-spatial model ideally requires four data points per patient. We are aware of the fact that many patients in the above-mentioned clinical trials have less than four data points available.

For all patients with four or more data points (optimal patient group), we can robustly fit the model (as our preliminary data show). For all patients with less than four data points (sub-optimal patient group), we need to investigate whether the model can uniquely and robustly fit the data. In this proposal, we request access to all patients, but possibly the final analysis will not include all sub-optimal patients.

## Sample size calculation

This is an exploratory, hypothesis-generating analysis that will yield predictions which can be validated in prospective clinical trials. Therefore, there cannot be any formal power analysis at this point.

We will use tumor volume measurements over time for individual patients in an anonymized way. We will then use these data to fit our existing computational models and analyze the robustness of the models in different types of diseases.

Please note that our models are implemented in Matlab and need to be run on our institution's computing clusters. Therefore, we have to download the following data to our own institution's computers:

- for each patient treated with anti-PDL1, we need tumor volume measurements over time

- in addition, if available, we will use the initial lymphocyte count as measured in biopsies

- the ultimate clinical outcome for each patient (RECIST)

We do NOT require any other clinical covariates including sex, ethnicity, age or geographical region. We confirm that we can work with data de-identified by Roche according to the rules specified on the CSDR webpage "Anonymisation Standards" (https://clinicalstudydatarequest.com/Study-Sponsors/Study-Sponsors-Roche.aspx).
